# Segregating signal from noise through movement in echolocating bats

**DOI:** 10.1038/s41598-019-57346-2

**Published:** 2020-01-15

**Authors:** Mor Taub, Yossi Yovel

**Affiliations:** 1 Department of Zoology, Faculty of Life Sciences, Tel Aviv University, Tel Aviv, 6997801 Israel; 2 Sagol School of Neuroscience, Tel Aviv University, Tel Aviv, 6997801 Israel

**Keywords:** Sensory processing, Animal behaviour

## Abstract

Segregating signal from noise is one of the most fundamental problems shared by all biological and human-engineered sensory systems. In echolocating bats that search for small objects such as tiny insects in the presence of large obstacles (e.g., vegetation), this task can pose serious challenges as the echoes reflected from the background might be several times louder than the desired signal. Bats’ ability to adjust their sensing, specifically their echolocation signal and sequence design has been deeply studied. In this study, we show that in addition to adjusting their sensing, bats also use movement in order to segregate desired echoes from background noise. Bats responded to an acoustically echoic background by adjusting their angle of attack. Specifically, the bats in our experiment used movement and not adaptation of sensory acquisition in order to overcome a sensory challenge. They approached the target at a smaller angle of attack, which results in weaker echoes from the background as was also confirmed by measuring the echoes of the setup from the bat’s point of view. Our study demonstrates the importance of movement in active sensing.

## Introduction

The problem of segregating signal from background is general for all sensory systems. Animals have evolved different strategies to optimize sensory acquisition and to deal with this problem^1–5^. Some of these strategies are at the level of the sensors or the neurons^6,7^, while others rely on active behavioral adjustments that aim to improve the signal-to-noise-ratio (SNR). Bats are considered masters of active sensing, constantly emitting echolocation sound signals to sense their environment, and adjusting their signals according to input and task^8–15^. Many bats are insectivorous and often hunt in or near vegetation. Searching for small prey items such as insects near large reflective surfaces such as vegetation creates a serious problem of segregating signal from background, as the echoes reflected from the background are typically several times louder than the desired signals. Bats’ ability to adjust their sensing, specifically the design of the echolocation signal and of the echolocation sequence has been studied extensively^8–12,16–20^. Such sensing modulations have been demonstrated in many bat species and include changing the parameters of the echolocation signals (e.g., signal duration and bandwidth), adjusting the sequence of signals (e.g., the inter pulse interval^12^) and modulating the beam’s direction^21^ and width^10,13^.

An alternative form of active sensing relies on movement to improve sensory acquisition^22^. Actuation of specific sensors such as eyes^23,24^, whiskers^2,25^ or ears^26–28^ are known to play an important role in enhancing sensory acquisition in many species. Recently it was shown that Carpenter ants use different patterns of antennae movement when following an odor trail^29^ in order to refine the information intake when faced with different levels of sensory difficulty. Improving sensory acquisition through movement of the entire body has also been demonstrated in many animals. Optic flow is a well-studied example of such a movement-dependent sensory strategy which has been shown to be used by bees^30^ and birds^31,32^. Several insects use movement of the entire body in order to allow visual-based depth perception^30,33^. Using this type of relative motion to solve the problem of signal from noise segregation was also shown in weak electric fish. These fish use back and forth movements in order to enhance detection via electrolocation^22,34^. Bats too have been shown to use various types of movement to enhance sensing. Some bat species use ear movements to improve directional sensing^27,35,36^. Bats that use constant-frequency echolocation use the prey’s wing-movement in order to detect prey in cluttered (i.e., highly echoic) environments^37^. A few studies suggested that bats also move differently depending on the type of background in their environment^38,39^, and that they might alter movement in order to improve SNR^40,41^ but so-far there was no consistent investigation of how bats use self-movement for segregating signal from noise and how movement based sensing interacts with adaptations of echolocation.

Our work was motivated by a study by Siemers and Schnitzler (2004) that showed that bats can capture tethered insects in close proximity to an echoic background^20^. The study emphasized the importance of the echolocation signal design for segregating echo from background, but it did not analyze the bats’ movement. The bats in that experiment often approach the target from the side, which led us to hypothesize that they were adjusting their movement in addition to adjusting echolocation. Specifically, we hypothesized that they will point their echolocation beam in a smaller angle relative to the background in order to reduce its echo. To test this idea we trained *Pipistrellus kuhlii* bats to search for and land on a platform while confronting them with different echoic situations. When faced with an acoustically echoic background (mimicking natural vegetation echoes), the bats adjusted their angle of attack in order to improve SNR as we also confirmed by recording the echoes of the target and the background. The bats did not adjust their echolocation parameters in order to improve SNR. Our results thus demonstrate the importance of movement in addition to echolocation for active sensing in bats.

## Results

**Figure 1:**
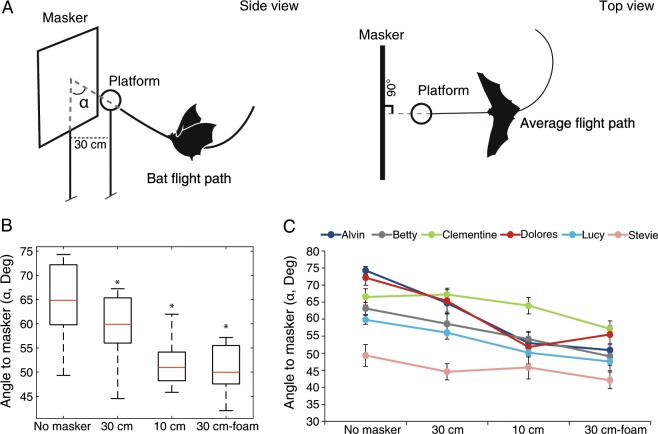
Changes in angle of attack relative to the masking board. (**A**) The angle of attack, α, is defined as the angle between the flight trajectory and the plane of the masking board (side view). The masking board was placed either 10 cm or 30 cm behind the platform. The masking board was positioned 90 degrees relative to the average flight path of each bat (top view). **(B)** Changes in α (degrees) for all bats in the different conditions, n = 6 bats. The whiskers indicate the extreme data points (beyond the percentiles) that are not considered outliers. Asterisks indicate a significant change in angle relative to either the no masker condition (for 30 cm and 10 cm) or to the 30 cm styrofoam condition (for 30 cm foam). **(C)** Changes in α in the different conditions for the six individual bats (different colors; mean ± SE). The same trend of decrease in angle can be seen in five out of the six bats. The sixth bat (Stevie) showed an initial decrease from no masker to 30 cm and then kept a relatively similar angle. Note that the initial angle in the no masker condition is already low relative to other bats and by decreasing it further in the masker conditions, this bat used the smallest angle in the experiment. The first bat, ‘Alvin’, performed an additional condition of 20 cm that corresponds to a different foam condition of 20 cm foam. This is because this bat did not have the 30 cm foam condition, and so in order to have a proper comparison between the conditions we compared the 20 cm styrofoam to 20 cm foam.

Six *Pipistrellus kuhlii* bats were trained to fly individually in a flight room and land on a spherical styrofoam target (15 cm diameter) where food was offered (henceforth the ‘no masker’ condition). Over a ~1 week training period, each bat developed a preference for a particular direction of flight from which it approached the target. After recording at least 40 (‘no masker’) landings for each bat, an acoustically masking board was placed either 30 cm (condition 1) or 10 cm (condition 2) behind the target. In order to induce maximal masking, the masking board was positioned at an angle of 90 degrees relative to the preferred horizontal 2D flight direction of each individual during training (i.e., the average flight path, Fig. 1A ‘Top view’). The masker was a 95 cm × 110 cm plastic board covered with plastic leaves, simulating a natural vegetation hedge (Fig. S1). The bats now had to deal with a problem of segregating a weaker signal (the target’s echo) from louder background noise - the echoes returning from the hedge-like masker. Echo recordings proved that at a distance of 100 cm from the masker, and consequently 70 cm from the platform, and at an angle of attack of 90 degrees (i.e., when directly facing the masker), the masker was 12 dB louder that the styrofoam target (Methods). To quantify the bats’ response, we monitored their flight and echolocation behavior.

Bats changed their flight pattern in response to the added masking (Fig. 1). To quantify the movement, we used the 3D angle of attack as we assumed that this angle should change if the bats were trying to improve SNR. We defined the 3D angle (α) as the angle between the bat’s acoustic gaze^17^, i.e., the direction of the echolocation beam, and a line on the plane of the masking board (see Fig. 1A and methods). All bats responded to the additional masking by significantly decreasing the angle of attack but different bats responded at different levels of masking. We ran all statistical tests both at the group and the individual level, including tests over all conditions and t-tests corrected for multiple comparisons as post-hocs (see Methods; The reduction in angle of attack was significant for both group and individuals - for the group: Generalized Linear Model, with condition as a fixed effect and bat ID as a random effect, F = 12.67,df = 4, P < 0.0001, n = 6 bats; For individual bats: One way ANOVA, F < 13.4, df = 4, P < 0.012 for all bats, see Table S1 for the bats P-values in all conditions).

We next analyzed the different conditions separately for both group and individuals. When the masker was 30 cm behind the styrofoam target, the group significantly reduced the angle of attack (α) by 5 ± 3 degrees on average (mean ± SD), and this response was significant in three of the six bats (Fig. 1B; P = 0.02 for the group, paired t-test with an FDR correction, n = 6 bats; P < 0.04, t-test with an FDR correction for the three bats that showed a significant response, Fig. 1C). When the task became more challenging, i.e., when the masker was only 10 cm behind the styrofoam target, the bats further decreased their angle of attack. The bats now significantly decreased the angle of attack by an average of 11 ± 7 degrees (mean ± SD) in comparison to the no-masker condition (Fig. 1B; P = 0.02 for the group, paired t-test with an FDR correction, n = 6 bats). In this condition, four out of the six bats showed a significant decrease relative to the no masker condition (P ≤ 0.001 t-test with an FDR correction, Fig. 1C). Two bats exhibited an overall significant response across all conditions (ANOVA test, see above) but although they both reduced the angle of attack (see pink and green lines in Fig. 1C) this response was not significant in the post-hoc tests for conditions 1–2. One bat (Stevie) always used the smallest angles of attack in comparison to other bats even when no masking board was present in the room (its average angle was 49 degrees with no masker in comparison to 67 degrees on average for the other bats, see pink line in Fig. 1C). When the masking board was placed 30 cm behind the platform, this bat decreased its angle to 44.5 degrees, a very low angle compared to the other bats in the same condition (which used an average angle of 62 degrees in this condition). It is probable that the initial small angle of attack displayed by this bat, in addition to this small decrease, were sufficient so that no further reduction was necessary in order to complete the tasks. The second bat that did not respond (Clementine) showed the same pattern of decreasing (α) as the other bats, but showed much variability and its decrease was not significant. This bat significantly responded by reducing the angle of attack when we switched to a foam target (see below). There were no consistent changes in the mean or maximum flight speed in the different sensory conditions (Table S2).

**Figure 2:**
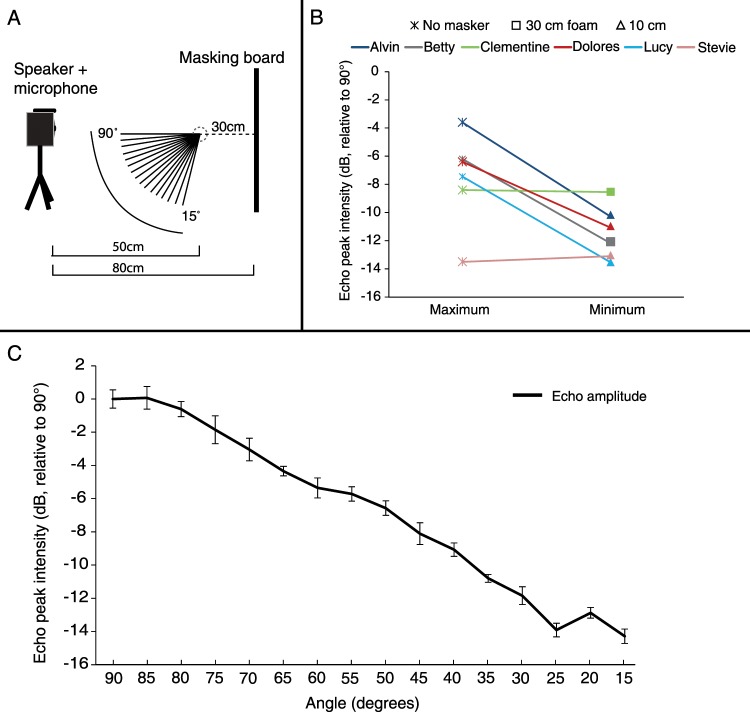
Ensonification of the masking board at different angles. (**A**) The set-up of the ensonification experiment. Bat-like signals were played through a speaker and returning echoes were recorded with a microphone located above the speaker. Recordings were performed at a distance of 50 cm from the platform with an additional 30 cm from the masking board (80 cm total), at sixteen angles. The platform was not present during the ensonification experiment. **(B)** The echo intensity for the smallest angles used by individual bats in the no masker condition (maximum) and the smallest angles used in any of the other experimental conditions (minimum). Colors indicate the different individuals and shapes indicate the condition in which the angle was minimal. **(C)** Echo peak intensity (dB) of the masking board at sixteen different vertical angles (mean ± SE). Echoes were band-pass filtered between 35–45 kHz. Intensities and SE are in logarithmic scale. Means were normalized relative to 90 degrees.

Decreasing the angle of attack as the bats did, significantly reduced the background masking echo intensity by an average of 3.8 ± 2.9 dB (mean ± SD for all six bats) and by 5.8 ± 0.7 dB (when excluding the two bats that did not respond; Fig. 2) as we revealed by measuring the intensity of the masker’s echoes at different angles of attack (see Methods; P = 0.03, paired t-test, n = 6 bats). Because the bats directed their echolocation beams straight towards the target (as we validated using tracking, see Methods and Fig. S2), and because the target was spherical, the intensity of the target’s echo should not have changed as a result of the smaller angle of attack. Therefore, the SNR should have increased by ~4–6 dB as a result of the change in flight pattern, and probably by more (see Discussion). However, even with this improvement in SNR, the masker was still louder than the target. Because the masker was 12 dB louder than the styrofoam target, a reduction of 4 dB in background intensity could not completely solve the masking problem.

To confirm that the observed movement adjustment resulted from a sensory interference and not from a difficulty to maneuver to the target in the presence of the masking board, we added another condition (condition 3). Here, the masking board was 30 cm behind the target (like in condition 1), but instead of the original styrofoam target, we used a foam sphere as a target (the foam target was 7 dB weaker than the styrofoam target while the diameter of the two targets was the same). This condition was the same as the first masking condition (condition 1) in terms of maneuverability (because the target and the masker were at the same distances) but it was sensory more challenging because the intensity of the target’s echo was reduced, while the intensity of the masker’s echo did not change. Bats significantly decreased their angle of attack by an average of 9 ± 3 degrees in this condition in comparison to condition 1 (mean ± SD Fig. 1B; P = 0.007 for the group, paired t-test with an FDR correction, n = 6 bats). Four out of six bats showed a significant decrease, including Clementine that did not respond significantly so far (P ≤ 0.03 for all four individual bats, t-test with an FDR correction; Fig. 1C). One bat (Alvin) showed the same pattern of angle reduction, but was not significant (P = 0.15). The order of the experimental conditions was as described above (no masker, 30 cm, 10 cm and 30 cm foam). However, in order to validate that the bats did not simply maintain the small angle of attack in condition 3 (30 cm foam), because they were used to it in condition 2 (10 cm), we first trained the bats to land on the foam platform (without a masking board) for four days. Indeed, in this situation, the bats increased their angle of attack (to an average of 57 degrees in comparison to 53 in condition 2). When the masker was then added (condition 3) the bats significantly reduced their angle of attack relative to this no masker foam-target control (P = 0.046 for the group, paired t-test with an FDR correction, n = 6 bats; three out of six bats showed a significant decrease, P ≤ 0.04, t-test with an FDR correction).

**Figure 3:**
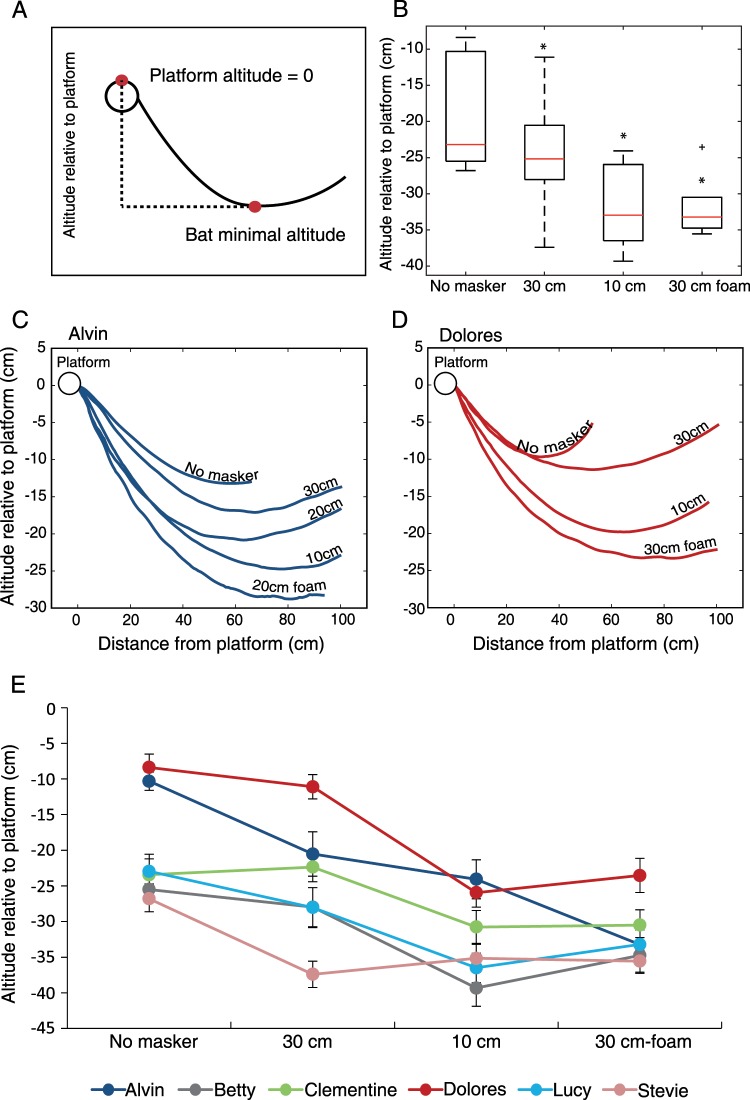
Minimal altitude of flight relative to the landing platform. (**A)** The minimal altitude was measured relative to the height of the platform which was normalized to zero. **(B)** The minimal altitude of flight (cm) relative to the platform for all bats in the different conditions, n = 6 bats. The whiskers indicate the extreme data points (beyond the percentiles) that are not considered outliers. Plus symbols indicate outliers (see MATLAB for outlier definition) and asterisks indicate a significant change in altitude relative to either the no masker condition (for 30 cm and 10 cm) or to the 30 cm styrofoam condition (for 30 cm foam). **(C**,**D)** Altitude along the flight path. Each line represents the average trajectory of each condition. The circle represents the location of the landing platform (at 0,0). Note the increase in the vertical curvature as sensory conditions become more difficult. Examples are shown for two of the six bats. **(E)** The minimal altitude of flight in the different conditions for the six individual bats (different colors; mean ± SE). The same trend of decrease in altitude can be seen in five out of the six bats. The sixth bat (Stevie) showed an initial decrease from no masker to 30 cm and then kept a relatively similar altitude. The first bat, ‘Alvin’, performed an additional condition of 20 cm that corresponds to a different foam condition of 20 cm foam. This is because this bat did not have the 30 cm foam condition, and so in order to have a proper comparison between the conditions we compared the 20 cm styrofoam to 20 cm foam. All other conditions are the same.

Because we calculated the angle of attack in 3D, the movement adjustment that we observed could be theoretically achieved by a horizontal decrease, i.e., increasing the horizontal curvature of the flight and approaching the target more laterally, or by increasing the vertical curvature, approaching from a lower altitude. We found that the bats mostly used the second option, dropping lower below the target when the task was sensory challenging (Figs. 3 and S3). Bats significantly decreased their minimal altitude below the target in all conditions, decreasing it more when the sensory challenge increased (Fig. 3B; P = 0.04 for no masker vs. condition 1, P = 0.002 for no masker vs. condition 2 and P = 0.03 for condition 1 vs. condition 3, paired t-test on the group level with an FDR correction, n = 6 bats). In the individual tests (Fig. 3C–E) three out of six bats flew significantly lower in condition 1 relative to the no masker condition (P ≤ 0.04, t-test with an FDR correction); All six bats flew significantly lower in condition 2 in comparison to the no masker condition (P ≤ 0.04, t-test with an FDR correction) and five out of six bats flew significantly lower in condition 3 in comparison to condition 1 (P ≤ 0.04, t-test with an FDR correction). Note that decreasing the vertical angle of attack does not necessarily mean lowering the flight trajectory. For example, the bats could reduce the angle of attack by flying at the same height but reaching the lowest part of their trajectory closer to the target.

If at all, there was a very weak adjustment of the horizontal angle of attack - the 2D horizontal angle defined between the bat’s emission direction and the plane of the masking board (Fig. S4). There was no significant reduction in horizontal angle of attack for the different conditions (for the group - Generalized Linear Model, with condition as a fixed effect and bat ID as a random effect, F = 3.15, df = 3, P = 0.056, n = 6 bats; Fig. S5).

Echolocating bats are renowned for their flexible sensing. Many studies have shown how bats adjust sensing to the task they are performing and to the environment in which they are performing it^8–12,17–20^. We thus tested if bats adapted their echolocation when the sensory difficulty increased (echolocation signals were recorded throughout the experiments with a microphone located on the landing target). We measured the signal duration, peak intensity and inter-pulse interval (IPI) of the echolocation signals (Figs. S6 and S7), three parameters that are routinely adjusted by bats, as well as the peak and terminal frequency. Bats did not seem to adapt their echolocation in order to solve the masking problem: no consistent change was observed in signal duration, IPI, peak frequency or terminal frequency within or between the conditions. There was no significant change in signal duration across conditions (Fig. S7A, repeated measures ANCOVA, with condition and distance from platform as factors and bat ID as random effect. P = 0.1, F = 2.08, df = 3). There was a significant difference in the inter-pulse intervals between conditions (Fig. S7B, repeated measures ANCOVA, with condition and distance from platform as factors and bat ID as random effect. P = 0.04, F = 2.86, df = 3). However, the change was minor and probably resulted from the strength of the target’s echo and not from the background masker as we observed a slightly higher IPI when using a foam target (pairwise ANCOVAs revealed a difference between condition 3 (30 cm foam) and condition 2 (10 cm), P = 0.01, F = 6.6, df = 1, as well as between condition 3 and the no masker condition, P = 0.02, F = 6.02, df = 1, but not for the other comparisons). Moreover, there was no significant change in peak or terminal frequency across conditions (Fig. S7C,D, repeated measures ANCOVA, with condition and distance from platform as factors and bat ID as random effect. For peak frequency P = 0.44, F = 0.91, df = 3; For terminal frequency P = 0.17, F = 1.7, df = 3). Bats also adjusted their signal intensity according to the echo strength of the reflecting objects - they reduced emission intensity when the background was louder, as has been shown before in echolocating bats^9^ (Fig. S7E, repeated measures ANCOVA with condition and distance from platform as factors and bat ID as random effect. P < 0.0001, F = 9.03, df = 3).

## Discussion

Insectivorous bats forage in diverse environments. In many species, the same individual can sometimes forage in open spaces with no or few background echoes and other times forage in highly echoic environments such as dense vegetation. Moreover, even bats that only forage in open spaces, often roost in caves or crevices, and thus must routinely deal with highly echoic environments. In all of these situations, bats must be able to detect prey, obstacles and landing locations, a task that becomes very difficult when moving in highly echoic settings in which the background echoes are often louder than the target’s echoes^11^. Bats’ ability to adjust their sensing, and specifically their echolocation signal and sequence design, has been extensively studied^8–12,16–20^, but in this study we show that they prefer to use movement in order to segregate desired echoes from background noise. When faced with an acoustically echoic background (mimicking vegetation echoes) the bats adjusted their angle of attack in order to improve the SNR. As the acoustic task became more challenging, because background-echoes became louder or because the target’s echoes became weaker, bats decreased their angle of attack relative to the masker thus emitting their sound beams in a smaller angle of attack relative to the background. When a sound wave impinges on a surface at smaller angles, more of the sound will be reflected onwards resulting in a weaker echo returning to the bat as we confirmed experimentally. If the surface was completely smooth, all of the energy would be reflected away from the bat. In the case of a hedge like the one that we used, reflectors are situated in many angles relative to the bat and thus much of the sound is reflected back to the bat. However, because the leaves’ orientation was far from uniform and most leaves were roughly vertical (Fig. S1), emitting sound in smaller angles reduces the intensity of the reflected echo. Changing the angle of attack, as the bats did, thus led to a reduction of ~4 dB (on average) in the intensity of the background echoes. Since bats directed their echolocation beam towards the spherical target (Fig. S2), its returning echoes should have maintained their intensity, and therefore, by changing the angle of attack, bats could increase SNR by at least ~4 dB.

Why did bats adjust the vertical curvature of the flight path more so than the horizontal curvature? We hypothesize that the bats mostly adjusted the vertical curvature because they were engaged in a landing task in which bats typically approach the target from below (even when there is no masker). Had they been tested in a task of catching tethered insects, we predict that they would have changed the horizontal curvature of the angle of attack (as the bats in the Siemers & Schnitzler 2004 study did), since kuhl’s pipistrelles typically catch tethered insects from the side, at least in the lab. The task of catching an insect is more demanding than landing; however, the great majority of behavioral experiments in the lab use tethered insects, in which case the task is not that different from a landing task. The target strength of our foam sphere was −52 dB which is equivalent to a large moth. Testing this response with a moving insect would be very interesting but also very difficult to perform.

Even after a reduction of ~4 dB in background intensity, the masker should have still been on average ~8 dB louder than the target, so how did bats overcome sensory masking? At least part of the solution for the masking problem lies in the temporal domain, as the masking echo returned to the bat’s ears between 0.6–1.8 ms after the target’s echo (equivalent to 10–30 cm), and the bats’ emission duration at the final stage of the approach was ~0.6 ms long (Fig. S7A). The background echo thus always returned to the bat after the target’s echo was received and it thus resulted in backward masking. There is no clear quantitative framework to calculate exactly how backward masking depends on time and intensity (see e.g., Blauert^42^), but it is likely that the combination of SNR improvement and time delay was enough to solve the sensory problem.

Why didn’t the bats fly even lower to further improve SNR? Flying lower probably has an energetic cost and in nature it may include an increased risk of collision or of predation, so the 3–4 dB additional improvement in SNR (see Fig. 2) was probably not worth the cost (one can hypothesize that the original flight trajectory is nearly optimal in terms of energy saving). We hypothesize that if the target was even closer to the background or smaller (and thus generated weaker echoes) the bats might have flown even lower. In addition, since the target was always located in the same place, it is possible that spatial memory played a role and that without it (e.g., if we moved the object in the room) the reaction would have been stronger. The bats could also reduce the angle of attack by reaching the minimal altitude closer to the target, but this would then probably elevate the costs of maneuvering to land.

The bats in our experiment used movement and not sensory acquisition in order to overcome a sensory challenge (we confirmed that the challenge was sensory using the foam target condition). Why did bats adjust movement and not echolocation to deal with masking? The task performed by the bats did not change in the different treatments. In all cases, they had to detect and localize the landing target and guide their flight accordingly. Bats typically adjust their echolocation parameters based on the distance of the target^16^ which did not change in the different conditions. Their echolocation parameters observed in the absence of the masker (e.g., signal duration and repetition), were probably already suitable for this task and thus did not change in the presence of background echoes. In theory, shortening the signals would reduce the masking temporally, but the bats were already using very short signals, probably close to their physiological limit. The bats did decrease the intensity of their calls when faced with the masker, but a decrease in calling intensity does not improve SNR as both target and background echoes will be weaker as a result of this behavior and the SNR will not change. Decreasing the call intensity is a well-documented behavior^9^ that probably aims to maintain the echo level within a certain dynamic range, that is, they adjusted echolocation so that the neuronal processing is maintained within a preferable dynamic range. Indeed, there was no significant reduction in calling intensity between the 10 cm and the 30 cm conditions (pairwise ANCOVA P = 0.3, F = 0.8, df = 1), strengthening the conclusion that this behavior is not related to SNR. Therefore, adjusting echolocation would not constitute a solution for the signal segregation problem, but by changing their movement strategy they managed to (at least) partially overcome masking. Naturally, the bats might have altered the different aspects of neural processing in the brain under the different conditions. Bats are renowned for their active sensing abilities, constantly adjusting their echolocation according to their momentary task. We therefore tend to forget the limits of echolocation, but this study demonstrates that other degrees of active sensing must always be taken into consideration.

## Materials and Methods

### Animals

Six female *Pipistrellus kuhlii* bats were captured according to permits from the Israeli National Park Authority (number 2016/41421). Bats were housed at Tel Aviv university’s Zoological gardens in a reversed light-dark cycle at 23–26 °C. Experimental protocols and procedures were approved and performed according to the Institutional Animal Care and Use Committee operating according to the Israel Health Ministry (ethics approval number: L-15-046). All methods were performed in accordance with the relevant guidelines and regulations.

### Behavioral experiments

Each individual bat was flown in the dark in a 5.5 × 4.5 × 2.5 m large flight room with acoustic foam on the walls and ceiling. Bats were trained to land on a 15 cm diameter sphere mounted on a 110 cm high wooden pole where mealworms were present. The landing sphere was made of either highly reflective styrofoam or of weakly reflective foam according to experimental condition (see below). The target remained at the same location in the center of the room throughout the experiment, to allow the bats to develop a stereotypic approach flight pattern. After ~1 week of landing without any disturbance, an acoustically reflective board (the masking board, Fig. S1) was added behind the target, perpendicular to it, ca. 90 degrees relative to the average horizontal 2D flight path of each bat (see Fig. 1A). The masking board consisted of a 95 cm × 110 cm plastic board mounted on a wooden pole and had plastic (artificial) vegetation connected to it sporadically to simulate natural clutter. The center of the masker was at the height of the landing target. Bats were recorded in three conditions in the following order: (1) Condition 1 - styrofoam sphere target with masking board positioned 30 cm behind the center of the sphere; (2) Condition 2 - styrofoam sphere target with masking board positioned 10 cm behind the center of the sphere, and (3) Condition 3 - foam sphere target with masking board positioned 30 cm behind the center of the sphere. In between conditions 2 and 3, the bats were first acclimatized to landing on a foam target without any masking (the training was the same as in the first week before the masker was introduced, but with a foam target). We analyzed the flight and echolocation behavior in these conditions as well as in the initial training to assess the bats’ response to the clutter.

### Tracking and audio recordings

Tracking was performed with a Motion Analysis Corp system. Up to twenty cameras (16 Raptor 1280 × 1024 pixels cameras and 4 Raptor-12 4096 × 3072 pixels cameras) were used to track the bats at a frame rate of 200 fps. Three spherical reflectors (2.4 mm diameter, 3 × 3 Designs Corp.) were glued to the bats using latex surgical cement (PERMA-TYPE, the PERMA-TYPE company Inc.). Reflectors were mounted on the center of the head in a T shape (two reflectors were slightly elevated relative to the third one, see Fig. S2B) allowing us to track the direction of the head in space (pitch and azimuth). Previous experiments confirmed that this system was able to track a moving reflector with an accuracy of ~1 mm and to track angle with an error SD of 0.8 degrees^43^. Audio recordings were performed using an ultrasonic wide-band microphone (USG Electret Ultrasound Microphones - Avisoft Bioacoustics/Knowles FG) connected to Hm1216 AD converter sampling at a rate of 375000 Hz. The microphone was connected to the landing target (at the connection point of the sphere and the pole) facing the direction where bats arrived from.

To correlate between the horizontal direction of the echolocation beam and the head’s direction we measured beam and head directions. Beam direction was measured with twenty-two ultrasonic wideband microphones (USG Electret Ultrasound Microphones - Avisoft Bioacoustics/Knowles FG) connected to two Hm1216 AD converter (Avisoft) which were synchronized by injecting an SMPTE code (Horita) into the least significant bit of their first channel. Eighteen microphones were evenly spread around the circumference of the room (100 cm between each two microphones) at a height of 120 cm, three microphones were located at 60 cm and one microphone was placed on the landing target (For detailed information about the beam analysis see Kounitsky *et al*.^10^). The head’s direction was measured with the tracking system (for detailed information see Eitan *et al*.^43^). Audio recordings were synchronized to the video tracking (Motion Analysis, inc).

### Echo intensity measurements

In order to simulate the echoes received by the bats and estimate their intensity, we ensonified the masking board and the target from different angles. We transmitted a 2 ms FM linear chirp (sweeping from 125 to 0 kHz) using a Vifa speaker connected to a DA converter (Avisoft player) and recorded the returning echoes with a calibrated microphone (GRAS, 40DP) connected to an HM116 AD converter at a sample rate of 375000 Hz. Echoes were recorded at a distance of 80 cm from the masking board, simulating the situation where the bat was 50 cm from the target and the masking board was 30 cm behind it (the target was not present during the ensonifications). This procedure was repeated for 16 vertical angles ranging from 15 to 90 degrees in 5 degrees intervals. Recorded echoes were band-pass filtered in Matlab (between 35–45 kHz) in order to account for *P.kuhlii’s* relevant frequency range. The average peak intensity of 45 echoes at each angle was taken as an estimate for echo intensity (and the standard deviation was also estimated). In addition, the two different targets (styrofoam and foam) were ensonified at an angle of 90 degrees (i.e., directly on-axis) from a distance of 100 cm. The peak echo of each recording was extracted. We could not ensonify both target and masker together reliably because the beam of the Vifa speaker is much narrower than that of a bat and the sphere thus occludes a substantial part of the masker. To measure the masking reduction resulting from the movement of each individual bat, we measured the difference in echo intensity between the smallest angle used by the bats in the no masker condition and the smallest angle used in other experimental conditions. We used the smallest angle as this should provide best SNR which in case of a sensory task is the important criterion. However, when performing the same analysis with the average angle the results remain the same (the average for all bats changes from 3.8 ± 2.9 dB for smallest angles to 3 ± 1.2 dB for the average angle).

### Angle calculation

The three dimensional angle (α) between the bats’ emission direction and the plane of the masking board was calculated for the final 50 cm of flight before landing (see Fig. 1A). During this part of the flight the bats were always flying straight towards the target so we could use their flight direction as a proxy for their acoustic gaze (we validated that bats pointed their head towards the target, see below). To estimate alpha, we took the smallest 15% of the angles within the last 50 cm of the flight and calculated their median. The same procedure was performed to estimate the 2D horizontal angle: the 2D angle between the bat’s emission direction and the plane of the masking board, parallel to the X-Y plane (see Fig. S4). In order to calculate the horizontal angle in the ‘no masker’ condition we used the position of the plane that was placed in the following conditions, since there was no plane in this condition (as a result some of the flights were parallel to the masking board. Any flight with an angle <10 degrees was excluded). For the 3D angle, we used a virtual plane for the calculation. Angle analysis was performed in Matlab (Mathworks, 2017b).

We validated that bats were always pointing their head towards the target during the flight (even when not flying directly towards it). Horizontal head direction was defined by the angle formed between the two head reflectors and the target (see Fig. S2A,B). Vertical head direction was defined by the angle formed between the top and bottom markers (mid-point between the top markers and the bottom marker) and the target. We ran the same calculation using the masking board instead of the target, taking the top point along the vertical mid-line of the board. Since the setup of the current experiment did not allow an accurate measurement of the emitted beams (because the masking board occluded many of the microphones in our room) we used the direction of the head of each individual as an estimate of its beam direction. We validated that there is a strong correlation between echolocation beam direction and head direction by measuring beam and head directions of five bats (Pearson R = 0.91, Fig. S2C). Since there was a strong correlation between the two, we could assume that head direction and beam direction were the same. There was no significant difference between head direction in the different conditions (P ≥ 0.9 for horizontal angle to masking board, P ≥ 0.5 for vertical angle to masking board; P ≥ 0.09 for horizontal angle to platform, P ≥ 0.09 for vertical angle to platform, paired t-test, n = 6 bats). Head direction remained steady along the flight path in individual trials as well. The head angles relative to the target ranged between 1–7 degrees on the horizontal axis (Fig. S2D,E), and 14–19 degrees on the vertical axis, suggesting that bats were directing their gaze at the target.

### Echolocation analysis

Signal parameter extraction was performed in Batalef; a Matlab based in-house software created for acoustic analysis. For each echolocation sequence, signals were detected automatically and then manually scrutinized to remove false detections. We then extracted five parameters often adjusted by echolocating bats in a task dependent manner: signal duration (defined −12dB relative to the peak), inter-pulse interval (defined as the time between the start of one signal and the start of the consecutive signal), peak frequency (frequency with most energy), terminal frequency (defined −12dB relative to the peak) and peak intensity. Parameters were measured from the envelope of the time signal.

### Statistics

To test the effect of the different conditions on the 3D and the horizontal angle of attack and the altitude of the flight, a generalized linear model (GLM with least squares method) was used for the group level, and a one-way ANOVA was used for each individual bat. Next, post-hoc pairwise comparisons between conditions were performed using paired t-tests for the group analysis and non-paired t-tests for individual bats. t-tests were used despite the small sample size (n = 6 bats) after consulting with an expert (Y. Benjamini^44^). To account for multiple comparisons, p-values obtained from t-tests were corrected by executing the Benjamini & Hochberg^45^ and the Benjamini & Yekutieli^46^ procedure for controlling the false discovery rate (FDR). All t-tests and one-way ANOVAs were performed in Matlab. GLM and Repeated measures ANCOVA analyses were performed in JMP software (SAS INSTITUTE Inc., USA).

## Supplementary information

Supplementary information.

## Data Availability

The datasets generated and analyzed during the current study are available at: https://www.dropbox.com/sh/met5cvcq9nmvxdd/AAAF4saT9FZl01FwWyRgD1pqa?dl=0
