# Supplementary material for: Segregating signal from noise through movement in echolocating bats
Source: Sci Rep. 2020 Jan 15;10:382. doi: 10.1038/s41598-019-57346-2 (PMC6962340; doi:10.1038/s41598-019-57346-2)
Supplement: Supplementary file 1 — Supplementary information. [file 41598_2019_57346_MOESM1_ESM.pdf]

Segregating signal from noise through movement in echolocating bats

Authors: Mor Taub[1] and Yossi Yovel[1,2*]

1. Department of Zoology, Faculty of Life Sciences, Tel Aviv University, Tel Aviv 6997801, Israel
2. Sagol School of Neuroscience, Tel Aviv University, Tel Aviv 6997801, Israel

Supplementary Information

	Condition/Bat	Alvin	Betty	Clementine	Dolores	Lucy	Stevie	Group
**Angle of attack**	One-way ANOVA (all conditions)	1.52E-08	1.89E-06	0.0005	1.96E-07	2.66E-05	0.0125	<0.0001 (GLM)
	No masker-10cm	<0.0001	0.0001	0.3561	<0.0001	0.0010	0.2930	0.0271
	No masker-30cm	0.0025	0.0584	0.7647	0.0398	0.0279	0.2034	0.0271
	30cm-30cm foam	0.1534	0.0001	0.0016	0.0326	0.0013	0.2930	0.0076
	No masker foam-30cm foam	0.0025	0.0584	0.2024	0.0398	0.0287	0.4681	0.0462
**Altitude**	One-way ANOVA (all conditions)	2.16E-07	3.02E-09	0.0048	8.88E-12	1.37E-08	3.99E-07	<0.0001 (GLM)
	No masker-10cm	<0.0001	<0.0001	0.0432	<0.0001	<0.0001	0.0001	0.0016
	No masker-30cm	0.0023	0.2578	0.6954	0.1779	0.0444	<0.0001	0.0454
	30cm-30cm foam	0.0154	0.0032	0.0096	0.0001	0.0444	0.2542	0.0326

Table S1: Corrected P-values for multiple comparisons. P-values obtained from t-tests were corrected for multiple comparisons by executing the Benjamini & Hochberg (1995) and the Benjamini & Yekutieli (2001) procedure for controlling the false discovery rate (FDR). P-values marked in blue indicate significance (below 0.05).

**Condition**	**No Masker**		**Condition 1 - 30cm**		**Condition 2 - 10cm**		**Condition 3 - 30cm foam**	
**Bat/Speed(m·s^{-1})**	**Avg**	**Max**	**Avg**	**Max**	**Avg**	**Max**	**Avg**	**Max**
**Alvin**	2	3.5	1.9	2.9	2	3.5	2	3.5
**Betty**	2.2	6.7	2.1	6	2.1	7.7	2.2	3.7
**Clementine**	2.3	5	2.2	4.2	1.9	3.3	2	3.5
**Dolores**	1.9	4.4	2	3.7	2.1	5.4	2	3.7
**Lucy**	2.3	3.6	2.2	4.1	2.3	3.8	2.3	4.9
**Stevie**	2.4	5.6	2.4	5.5	2.4	4.1	2.4	5

Table S2: Flight speed. Average and maximum speeds (m·s^{-1}) of each bat in the different conditions. There were no consistent changes in speed in the different sensory conditions.

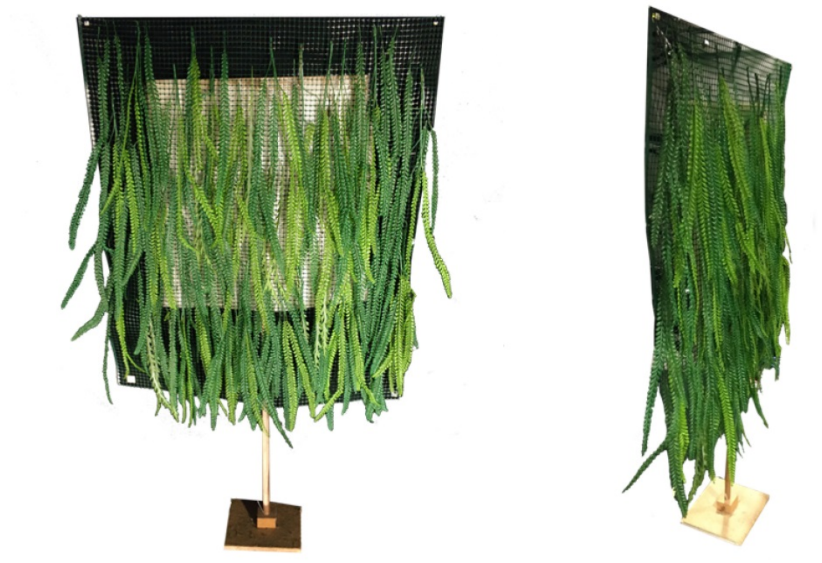

Figure S1: Masking board. The masking board was a 95cmX110cm plastic board mounted on a wooden pole and was covered with plastic (artificial) vegetation connected to it sporadically to simulate natural vegetation hedge.

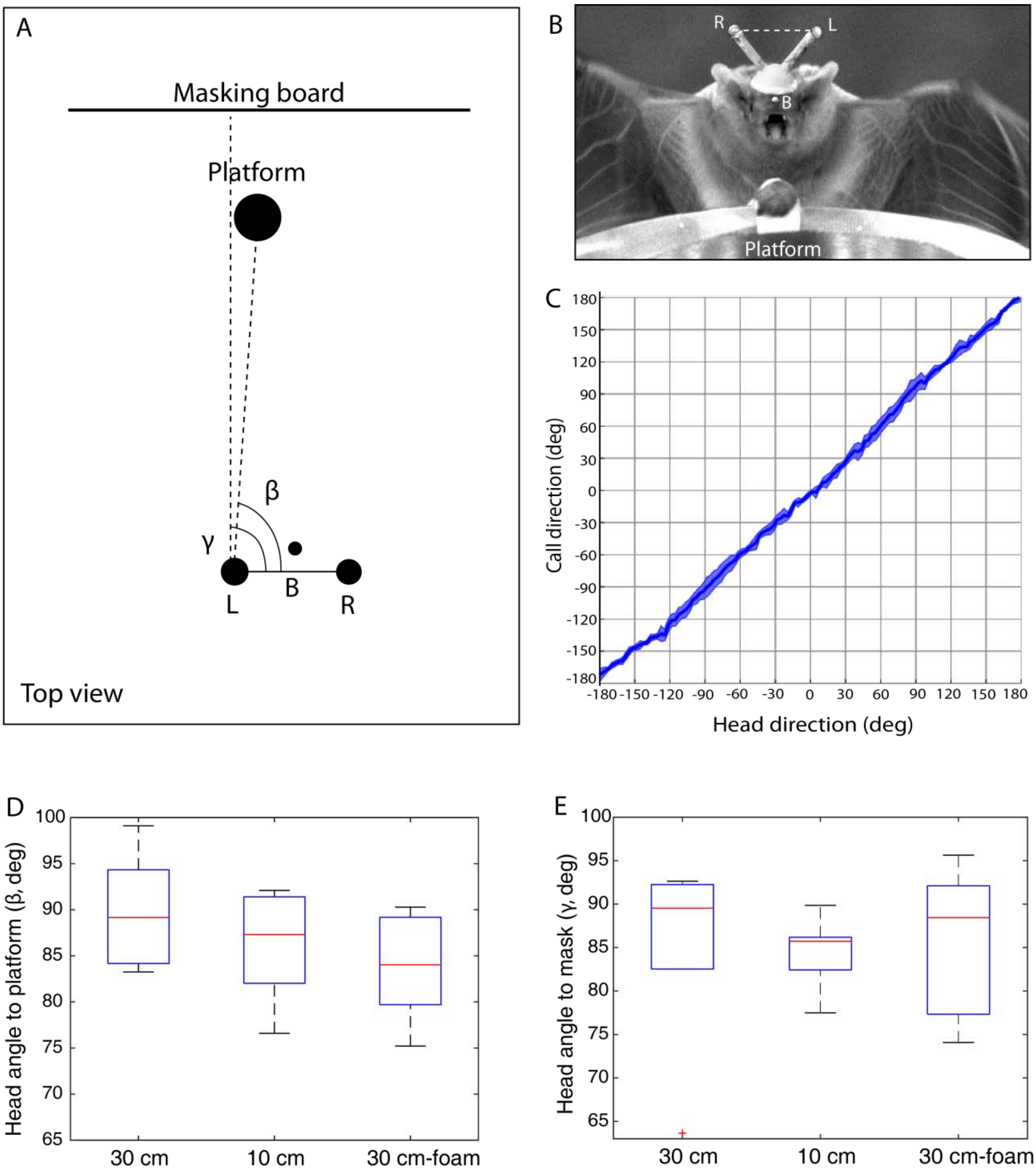

Figure S2: Head direction as an estimate of acoustic gaze. (A) Head direction was defined by the angle formed between the two horizontal head markers (left-L and right-R) and either the platform (β) or the masking board (γ). **(B)** The markers were placed on the bat's head in a T shape. Two markers (L,R) were elevated relative to the third (bottom-B). **(C)** There is a strong correlation (Pearson R=0.91) between echolocation beam direction and head direction. Beam direction was measured by recording bat signals with a 22 ultrasonic wideband microphone array (microphones were evenly spread around the circumference of the room). Head direction was measured with the tracking system (see full details in Eitan et al, 2019). The graph shows the average and STD for 5 bats. This correlation allowed us to estimate beam direction from the head direction. **(D)** Head angle to the platform (β) in the three conditions (Conditions 1,2,3). There was no significant difference between conditions ($P \geq 0.09$) with average angles ranging from 83 to 89 degrees. **(E)** Head angle to masking board (γ) in the three conditions. There was no significant difference between conditions ($P \geq 0.8$) with average angles ranging from 84 to 86 degrees.

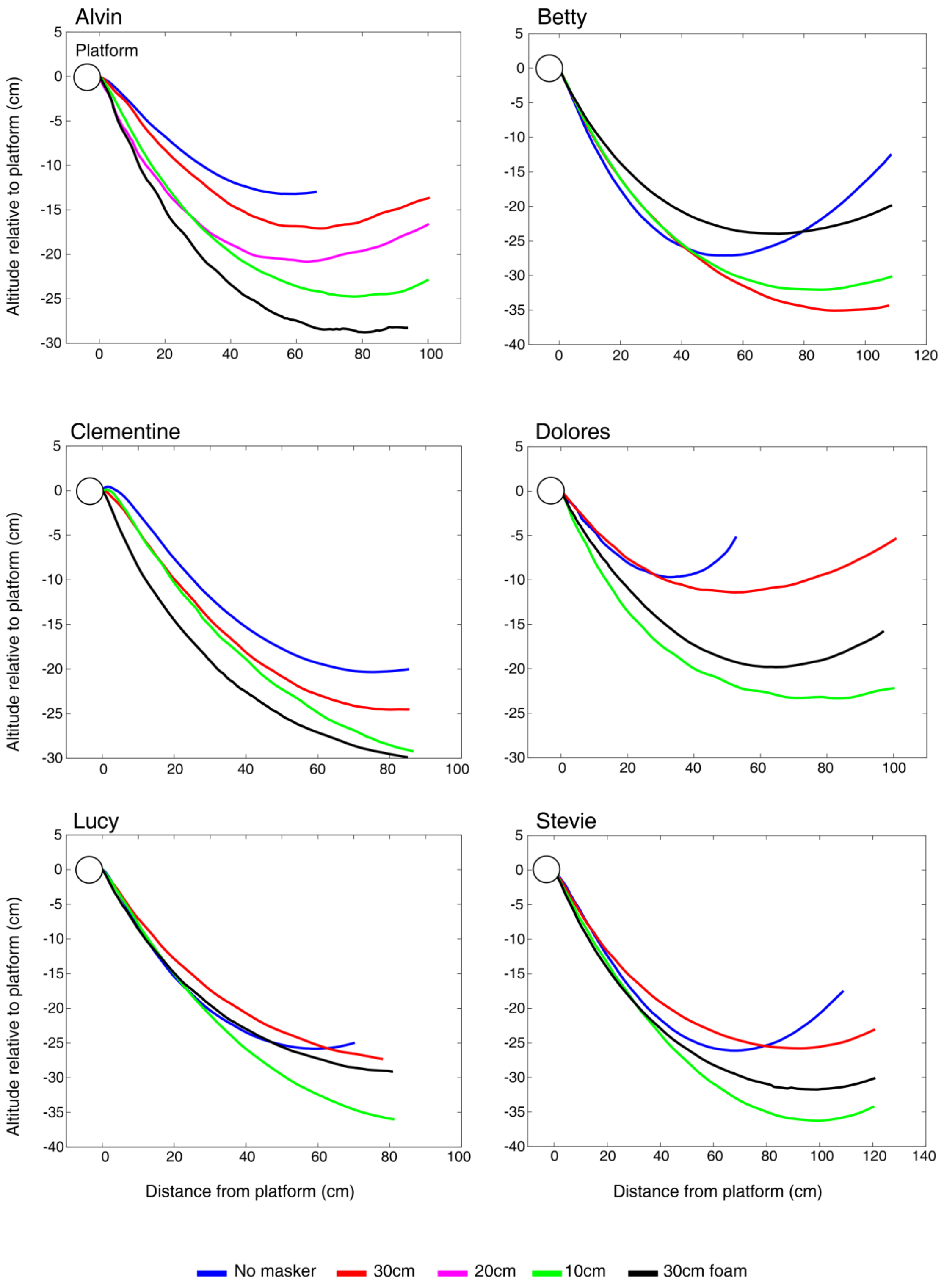

Figure S3: Z-axis flight paths as a function of distance from the platform. Flight altitudes in each condition (average per bat) are displayed on the vertical axis for each individual bat. Bats show a decrease in altitude as the sensory conditions become more difficult (different colors). Circles indicate the location of the landing platform (at 0,0). Note that axes are not equally spaced.

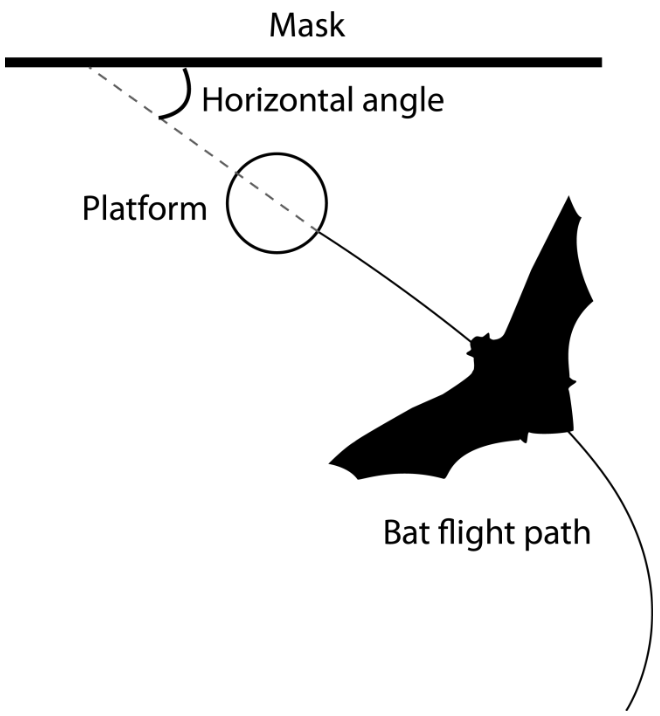

Figure S4: Horizontal angle of attack. The horizontal angle is defined as the 2D angle between the bat's emission direction and the plane of the masking board, parallel to the X-Y plane.

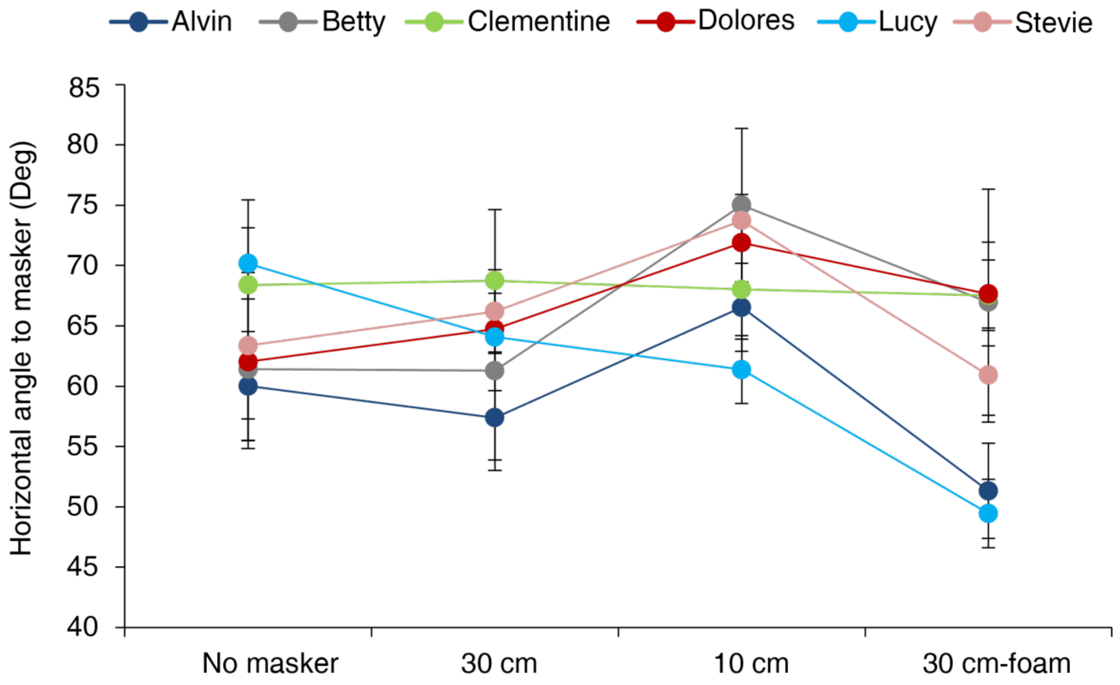

Figure S5: Changes in horizontal angle of attack relative to the masking board. Changes in horizontal angle of attack, in the different conditions for the six individual bats (different colors; mean±SE). There was no significant reduction in horizontal angle of attack for the different conditions. None of the bats significantly reduced this angle when the masker was introduced (no masker vs. condition 1, P=0.7 for the group, paired t-test with an FDR correction, n=6 bats. The angle remained at 64 degrees on average). When the masker was moved closer to the styrofoam platform, at 10cm, the average angle increased to 69 degrees with only one bat significantly reducing their angle (no masker vs. condition 2, P=0.04, t-test with an FDR correction. Two other bats showed a significant change but for both, the angle for condition 2 was larger than the angle for the no masker condition, thus contrary to expectation). When changing the material of the landing target (condition 1 vs. condition 3), one out of six bats showed a significant decrease in angle, in the right direction, (P=0.005, t-test with an FDR correction). And although there was no significant reduction in angle between condition 2 (masker 10cm ; 69 degrees on average) and condition 3 (30cm foam; 61 degrees on average) for the group (P=0.06 for the group, paired t-test with an FDR correction, n=6 bats), it seems that some of the bats did change their horizontal angle of attack (three out of six bats showed a significant decrease in horizontal angle, P<0.006, t-test with an FDR correction).

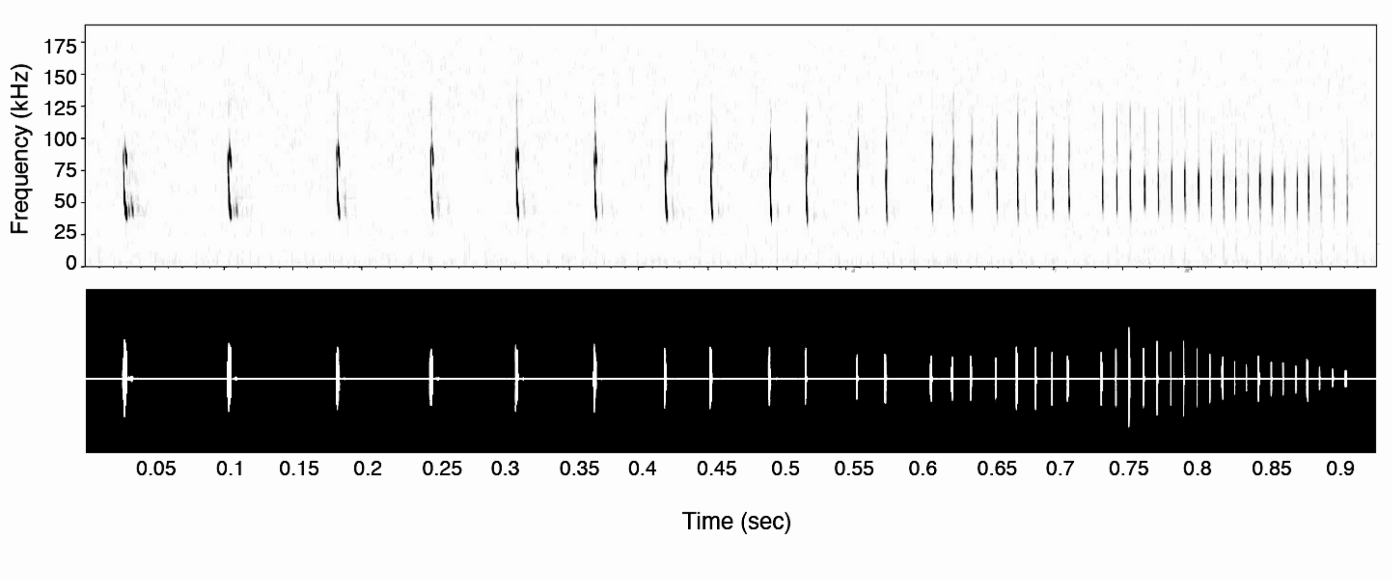

Figure S6: Echolocation sequence. Spectrogram (top) and time signal (bottom) of a flight echolocation sequence (from the no masker condition) ending when the bat lands on the platform.

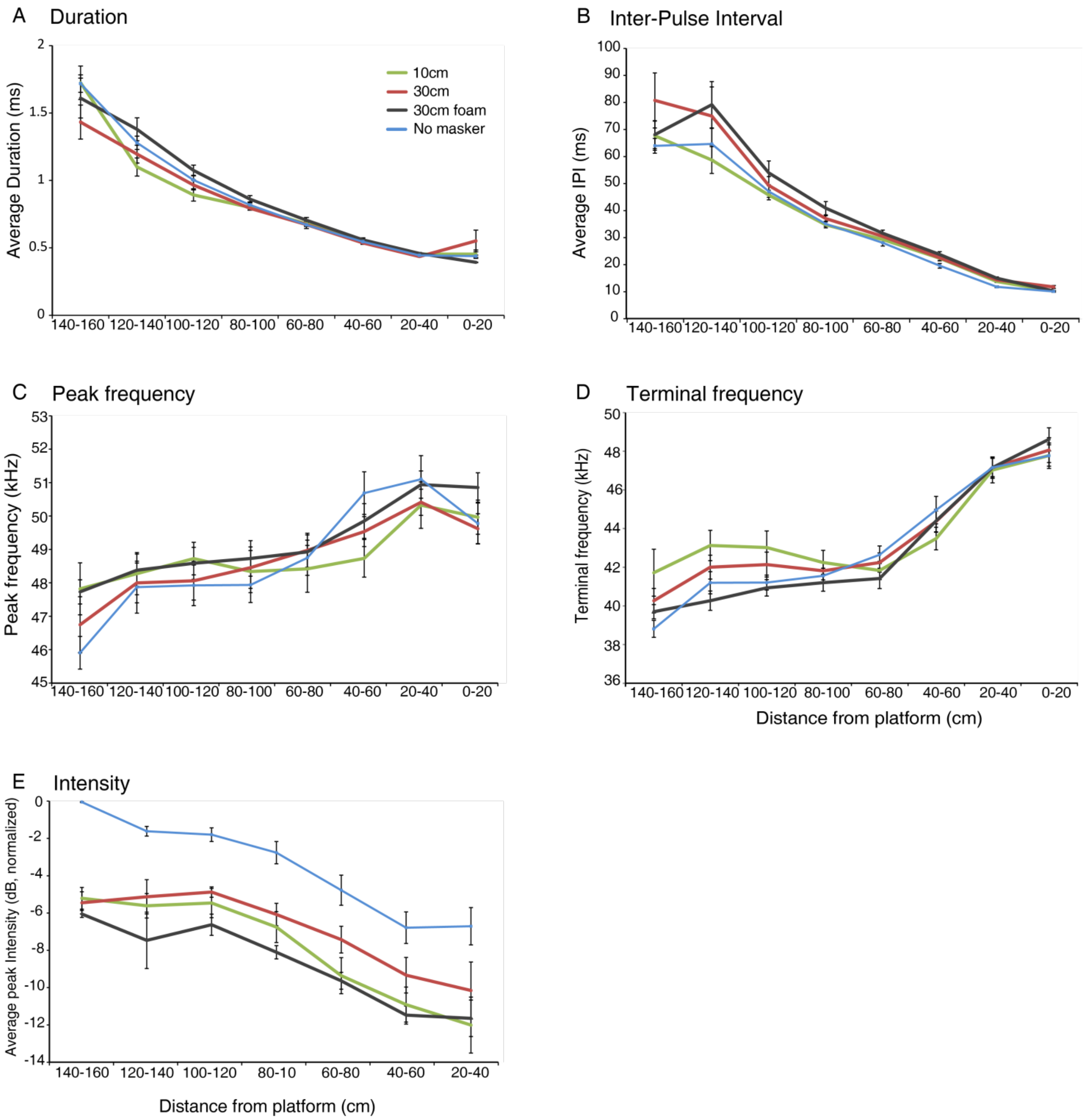

Figure S7: Echolocation signal parameters along the approach. (A) Duration decreased as the bats approached the platform (mean±SE) in all conditions (different colors). **(B)** Inter pulse interval (IPI) decreased as the bats approached the platform (mean±SE). There is a minor significant difference in IPI between the no masker and the 30cm foam condition as well as the 10cm and 30cm foam condition. However, the change probably resulted from the strength of the target's echo and not from the background masker as we observed a slightly higher IPI when using a foam target. **(C)** Peak frequency increased as the bats approached the platform (mean±SE). There was no significant change in peak frequency across conditions. **(D)** Terminal frequency increased as the bats approached the platform (mean±SE). There was no significant change in terminal frequency across conditions. **(E)** Intensity decreased as the bats approached the target (mean±SE) and was also lower for the masker conditions. Data for the distance of 0-20cm is not shown since at that distance bats are very close to the microphone and the intensity of signals is influenced by their relative landing spot on the target. For all parameters n=5 since one bat (Alvin) had insufficient data.
